# Sex differences in cancer incidence: prospective analyses in the UK Biobank

**DOI:** 10.1038/s41416-025-03028-y

**Published:** 2025-05-08

**Authors:** Maira Khan, Keren Papier, Kirstin L. Pirie, Tim J. Key, Joshua Atkins, Ruth C. Travis

**Affiliations:** Cancer Epidemiology Unit, Nuffield Department of Population Health, University of Oxford, Oxford, UK

**Keywords:** Risk factors, Cancer epidemiology

## Abstract

**Background:**

We examined differences in cancer incidence between women and men and the extent to which these persisted after accounting for established risk factors.

**Methods:**

Prospective analyses in the UK Biobank to examine associations between sex and risk of 15 cancers (and 13 subtypes) using minimal and multivariable-adjusted Cox proportional hazards regression models. Multivariable models were stratified for age, deprivation index, and region, and adjusted for ethnicity, qualifications, height, BMI, smoking status, alcohol, and site-specific risk factors.

**Results:**

During 10.5 (SD 2.2) years of follow-up, 32,315 incident cancers across 15 anatomical sites (58.1% in women) were identified in 470,771 individuals (53.8% women). Some differences in cancer risk between the sexes attenuated to the null in the multivariable-adjusted models, but men remained at greater risk than women for cancers at eight sites: oesophageal adenocarcinoma (hazard ratio 5.45; 95% confidence interval, 4.18–7.12), gastric cardia (3.65; 2.48–5.38), bladder (3.47; 2.85–4.24), oral cavity (2.06; 1.69-2.51), liver (1.91; 1.48–2.47), kidney (1.77; 1.51–2.09), rectum (1.70; 1.47–1.96), and leukaemia (1.43; 1.21–1.69). Men had lower risks for cancers of the breast, thyroid (0.36; 0.26–0.49), anus (0.41; 0.26–0.64), and lung adenocarcinoma (0.72; 0.62–0.84).

**Conclusion:**

Further research on these sex differences in risk may provide insights into cancer aetiology.

## Introduction

Most cancers common to both sexes exhibit some level of sex difference, with generally higher incidence and mortality of cancers at non-reproductive sites in men than women [1–4]. In men, the risk is typically 1.5 to 3 times greater, except for cancers of the anus, gallbladder, and thyroid, where the incidence is higher in women [4–6]. Cancers of the bladder, oral cavity, oesophagus, and liver have nearly threefold higher incidence in men [4–6]. Between 2016 and 2018, age-standardised incidence rates for all cancers among adults aged 35–69 in the UK (excluding the two sites with the largest incidence, breast and prostate) were 363.8 per 100,000 for women and 422.3 per 100,000 for men [7]. Over the last 25 years, these cancer incidence rates have initially declined and then stabilised in men, while a modest annual increase has been observed in women [7]. The cancer sites with rising incidence rates in both sexes include cancers of the oral cavity, liver, kidney and melanoma, along with lung cancer in women [7], all sites which exhibit well-documented sex differences [4–6].

The differences in cancer incidence between sexes have been mostly attributed to differences in exposure to established risk factors, which are largely anthropometric (height and obesity), lifestyle related (smoking, alcohol consumption, diet, and physical activity), molecular (chronic inflammatory and endocrinological risk factors) and genetic (EXITS genes, mosaic Y-loss) [3, 6, 8]. Statistics on sex differences in cancer incidence are usually reported on a national registry level, and they do not account for the hypothesised individual-level cancer risk factors [4, 5, 8, 9]. There have also been limited large-scale cross-cancer-site prospective cohort analyses of sex differences in cancer risk [6].

Exploring the underlying sex differences in cancer incidence and the potential reasons for the differences may provide insights into cancer aetiology and further research directions. Performing an outcome-wide analysis in one cohort allows analyses to be standardised across cancers. The aim of our study was to carry out the first comprehensive prospective analyses to examine the differences in incidence of cancers (at 15 shared anatomic sites and 13 subtypes) between men and women in the UK Biobank (UKB) and to assess the extent to which any disparities persisted after accounting for established and measured risk factors for those cancers.

## Methods

### Cohort design and baseline assessment

The UK Biobank is an ongoing cohort study of half a million participants (aged 37–73 years at recruitment) from the United Kingdom, registered with the UK National Health Service [10]. Participants residing in England, Wales, and Scotland volunteered at 22 assessment centres between 2006 to 2010, with a 5.5% response rate (6.4% women vs 5.1% men) [10, 11]. The study was approved by the North West Multi-Centre Research Ethics Committee (UK) (06/MRE08/65), the Patient Information Advisory Group (England and Wales), the National Information Governance Board for Health and Social Care (England and Wales) and the Community Health Index Advisory Group (Scotland) [12]. Participants joined by completing baseline touchscreen questionnaires, providing biological and anthropological data, and giving informed consent for their health to be followed up through electronic medical records [10]. Researchers can apply to use the resource and access the data used in our study via https://www.ukbiobank.ac.uk/enable-your-research/apply-for-access. Details on how the risk factors were measured are described in the supplementary information.

### Exclusion criteria

Participants were excluded if at recruitment they had missing information on sex, region, age, standing height, or BMI, or if they had any cancer (except C44: nonmelanoma skin cancer) prior to entry (*n* = 31,615). Supplementary Fig. 1 details the exclusion criteria.

### Exposure

Participants’ sex was acquired from their National Health Service (NHS) records at recruitment, but in some cases, this may have been updated based on self-reports (UKB data field 31). We further checked whether participants’ self-reported sex matched their genotyped sex (data field 22001 with two categories, female and male; available for 98.98% of participants). Participants were excluded if they had differences (367 participants) between their sex as recorded on the NHS record or self-reported at recruitment and their sex as determined by UK Biobank genotyping analyses.

### Assessment of cancer incidence

Cancer diagnoses were captured through linkage to national cancer and death registries, and the outcomes were defined as first incident cancer diagnosis or cancer first recorded in death certificates. All outcomes were defined according to the World Health Organization’s International Statistical Classification of Diseases (ICD-10). Cancer data and death data in England and Wales are provided by NHS England, and in Scotland by the NHS Central Register and the National Records of Scotland [13, 14]. The person-years of follow-up were calculated from baseline assessment at recruitment until the first registration of cancer, date of death, loss, or end of follow-up, whichever came first. The cancer data censoring dates were 31 December 2020 for England, 31 December 2016 for Wales, and 30 November 2021 for Scotland [13, 14].

The endpoints and their respective ICD-10 codes were: cancers of the lip, oral cavity, and pharynx [C00-C14] (oral cavity, hereafter), oesophagus [C15] with oesophageal adenocarcinoma and oesophageal squamous cell carcinoma, stomach [C16] with gastric cardia [C16.0] and gastric non-cardia [C16.1-C16.6], colorectum [C18-C20] with colon [C18] and rectum [C19-C20], anus [C21], liver [C22], gallbladder [C23], pancreas [C25], lung [C34] with lung adenocarcinoma, lung small cell carcinoma and lung squamous cell carcinoma, malignant melanoma [C43] (melanoma, hereafter), breast [C50], kidney [C64-C65], bladder [C67], thyroid [C73], and lymphatic and hematopoietic sites [C81-C96], with Hodgkin lymphoma [C81], non-Hodgkin lymphoma (NHL) [C82-C85], multiple myeloma [C82-C85], and leukaemia [C91-C95]. Breast cancer was included because, although this cancer is rare in men, there are some aetiological risk factors shared between men and women [15]. The histology codes used for distinguishing cancer subtypes are listed in Supplementary Table 1.

### Statistical analyses

Cox proportional hazards (PH) regression models with attained age as the underlying timescale were used to estimate hazard ratios (HRs) and 95% confidence intervals (CIs) for the risk of cancer in men compared to women, treating each cancer as a different endpoint. The analysis included 28 cancer endpoints (15 main sites and 13 subtypes) with at least 100 cases for each. First, a minimally-adjusted Cox model was used with stratification by age group at recruitment (<45, 45 to <50, 50 to <55, 55 to <60, 60 to <65, and ≥65 years), Townsend index of deprivation (quintiles; a score that incorporates census area data for employment, car ownership, home ownership, and household overcrowding [16]), and region (ten regions: London, North-West, North-East, Yorkshire and Humber, West Midlands, East Midlands, South-East, South-West, Wales, and Scotland). Second, a multivariable-adjusted Cox model was used, which additionally adjusted for qualification status (college or university degree/vocational qualification, A levels or equivalent (national examination at 17–18 years), O levels or equivalent (national examination at 16 years), and other/unknown qualifications), ethnicity (Asian or Asian British, Black or Black British, Mixed, Other, and White), height (continuous, measured in cm), body mass index (BMI; calculated as weight in kilograms divided by height in metres squared, <25, 25 to <30, 30 to <35, and ≥35 kg/m^2^), smoking (categorised by a composite variable that combined smoking intensity and status; never, former (<15 and ≥15 cigarettes/day), and current (<15 and ≥15 cigarettes/day)), and alcohol intake (categorised based on intake in grams per day; never drinkers, <1, 1 to <10, 10 to <20, 20 to <30 and 30+ g/day). Missing categories were generated for the following variables: qualifications (0.9% participants had missing data), ethnicity (0.5% missing), smoking (0.5% missing), and alcohol (0.6% missing). Categorical adjustment variables were used throughout to minimise any undue influence of extreme values, except for height for which a continuous variable was used because of the substantial non-overlap between women and men for this variable and because it was measured under standardised conditions and therefore there should be no erroneous extreme values. Details on how the risk factors were measured are described in the supplementary information.

Third, cancer site-specific covariates were added to the multivariable-adjusted Cox models. The covariates were selected based on the availability of data in the cohort, and on prior scientific understanding of risk factors for individual cancer sites, including through reference to a similar analysis performed in the National Institutes of Health-American Association of Retired Persons Diet and Health Study [6], and the Textbook of Cancer Epidemiology (3rd ed.) [17], which together provided the a priori rationale (described in Supplementary Table 2). Additional covariates included prevalent diabetes mellitus (for cancers of the gastric cardia, colorectum, colon, and rectum), inflammatory bowel disease and processed meat intake (for cancers of the colorectum, colon, and rectum), prevalent HIV and number of lifetime sexual partners (for cancer of the anus), alcoholic liver disease and non-alcoholic fatty liver disease (for cancers of the liver), gallbladder disease (for cancers of the gallbladder and liver), use of UV protection, time spent outdoors in the summer, and ease of skin tanning (all for melanoma), hypertension (for cancers of the kidney), and goitre (for cancers of the thyroid). Number of sexual partners was coded as a proxy for probable exposure to human papillomavirus infections [15]. For the cancer-specific multivariable adjusted analyses, we further excluded 51 participants from the cohort who had a history of gastrectomy at recruitment as these individuals had a lower risk of cancers of the pancreas (associated with complete gastrectomy) and stomach (partial gastrectomy) [18, 19]. Details on the International Classification of Diseases (ICD-10 and ICD-9) and operating procedure codes (OPCS-4) used for coding prevalent diseases and operations have been provided in Supplementary Table 2. Multiple testing was corrected for using the Bonferroni method (0.05/28 tests), with *p* < 0.0017 considered statistically significant.

Tests based on Schoenfeld residuals and log-log plots were used to check for violations of the proportional hazards assumption for each cancer-site-specific multivariable-adjusted Cox model. There was no evidence of significant violation of the proportional hazards assumption, except for breast cancer and melanoma. Violations of the proportional hazards assumption were not explored further for breast cancer due to the rarity of this cancer in men. For melanoma, the effect of sex varied with attained age (*p* < 0.0001), and so the multivariable-adjusted Cox model was rerun separately in those aged <65 years and those aged ≥65 years (threshold selected based on median age at melanoma diagnosis).

### Sensitivity analyses

Sensitivity analyses were performed on the cancer-site-specific multivariable-adjusted Cox models to assess residual confounding. First, the analyses were restricted to self-reported never-smokers at recruitment. Within this analysis, we additionally adjusted for exposure to second-hand smoke (for lung adenocarcinoma), which was measured by calculating the number of hours per week participants who identified as never smokers were exposed to tobacco smoke at home and outside home. Second, analyses were restricted to light drinkers i.e. participants who reported drinking <20 g/day at recruitment (not including participants who reported as never drinkers); the multivariable-adjusted Cox models also included adjustment for alcohol categories for this analysis. Third, we restricted the analyses to participants in the highest qualification attainment group (college or university degree/vocational qualification). Fourth, we additionally included a quadratic term for height, to allow for a non-linear effect. Fifth, we additionally adjusted for the effects of physical activity, classified by low (<10 excess metabolic equivalents (METS)/week), moderate (≥10 to <50 excess METS/week), and high (≥50 excess METS/week), with a separate category for missing values. All analyses were performed using STATA (version 18) and results were plotted using R (2023) [20, 21].

## Results

### Study participants

**Fig. 1:**
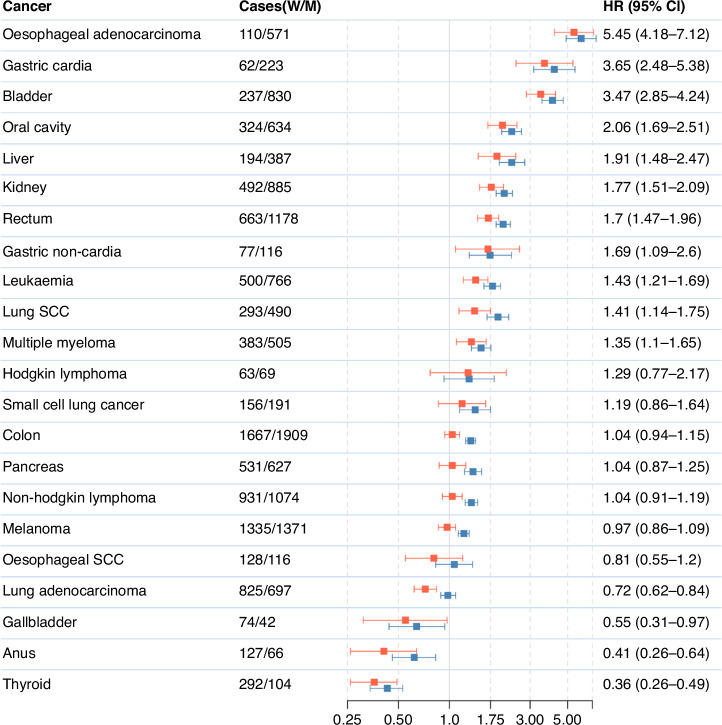
Sex differences in cancer incidence in the UK Biobank. Hazard ratios (HR) and 95% confidence intervals (CI) for incident cancer in men compared to women in UK Biobank, from minimally-adjusted (blue) and cancer-specific multivariable-adjusted Cox models (orange)*. M men; SCC squamous cell carcinoma; W women.

The final study cohort included 470,771 participants aged 37–73 years at recruitment, 253,344 (53.8%) women and 217,427 (46.2%) men (Table 1). On average, women were aged 56 (SD 8) and men were aged 56 (SD 8) at recruitment. During an average 10.5 (SD 2.2) years of follow up (10.6 (SD 2.1) years for women and 10.4 (SD 2.4) years for men), 32,315 incident cancers were identified at the shared anatomical sites; 18,774 cancers (58.1%) in women and 13,541 (41.9%) in men. Of these, the five most common cancers in men were colon, lung, melanoma, rectum, and non-Hodgkin lymphoma (Fig. 1 and Table 2). The five most common cancers in women were breast, lung, colon, melanoma, and non-Hodgkin lymphoma (Fig. 1 and Table 2).

**Table 1:** Baseline distribution of covariates and details of follow-up in the UK Biobank

	Women	Men	Total
Cohort size	253,344	217,427	470,771
Follow-up for cancer incidence			
Number of incident cancers	18,774	13,541	32,315
Person-years of follow-up, (100,000 s)	26.9	22.4	49.4
Mean follow-up time [SD]	10.6 [2.1]	10.4 [SD 2.4]	10.5 [2.2]
Median follow-up time [IQR]	10.9 [10.1–11.7]	10.9 [9.9–11.6]	10.9 [10.1–11.7]
Age, years			
Mean age at recruitment [SD]	56.1 [8.0]	55.6 [8.2]	56.3 [8.1]
<45	26,967 (10.6)	23,412 (10.8)	50,379 (10.7)
45–49	35,285 (13.9)	28,497 (13.1)	63,782 (13.5)
50–54	40,712 (16.1)	31,991 (14.7)	72,703 (15.5)
55–59	47,049 (18.6)	38,334 (17.6)	85,383 (18.1)
60–64	60,034 (23.7)	52,002 (23.9)	11,2036 (23.8)
>65	43,297 (17.1)	43,191 (19.9)	86,488 (18.4)
Region			
London	35,512 (14.0)	28,905 (13.3)	64,417 (13.6)
North-West England	38,656 (15.3)	34,894 (16.1)	73,550 (15.6)
North-Eastern England	29,488 (11.6)	25,358 (11.7)	54,846 (11.7)
Yorkshire and the Humber	37,506 (14.8)	32,334 (14.9)	69,840 (14.8)
West Midlands	21,284 (8.4)	20,695 (9.5)	41,979 (8.9)
East Midlands	17,194 (6.8)	14,690 (6.8)	31,884 (6.7)
South-East England	22,419 (8.9)	18,336 (8.4)	40,755 (8.6)
South-West England	22,314 (8.8)	18,111 (8.3)	40,425 (8.5)
Wales	10,431 (4.1)	8996 (4.1)	19,427 (4.1)
Scotland	18,540 (7.3)	15,108 (7.0)	33,648 (7.1)
Deprivation Index, in quintiles			
Most affluent	50,544 (19.9)	43,884 (20.2)	94,428 (20.0)
2nd	50,755 (20.0)	43,411 (19.9)	94,166 (20.0)
3rd	51,320 (20.4)	42,785 (19.7)	94,105 (20.0)
4th	51,234 (20.2)	42,724 (19.7)	93,958 (20.0)
Most deprived	49,197 (19.4)	44,340 (20.4)	93,537 (19.8)
Unknown^a^	294 (0.1)	283 (0.1)	577 (0.1)
BMI, kg/m^2^			
Mean BMI [SD]	27.1 [5.2]	27.9 [4.3]	27.4 [4.8]
<25	99,284 (39.2)	53,579 (24.6)	152,863 (32.5)
25–29.9	93,652 (36.9)	107,730 (65.8)	201,382 (42.8)
30–34.9	40,050 (15.8)	43,377 (26.5)	83,427 (17.7)
≥35	20,358 (8.0)	12,741 (7.8)	33,099 (7.0)
Height, cm			
Mean height [SD]	162.5 [6.3]	175.6 [6.8]	168.5 [9.3]
Qualifications			
Prof Q/NVQ/HND/HNC/Degree or other professional qualification	143,289 (56.6)	136,211 (62.6)	279,500 (59.4)
A Levels/or equivalent	14,694 (5.8)	10,922 (5.0)	25,616 (5.4)
O level/GSE/CSE/or equivalent	48,762 (19.2)	29,330 (13.5)	78,092 (16.6)
None of the above	41,864 (16.5)	36,663 (16.9)	78,527 (16.7)
Unknown^a^	4735 (1.8)	4301 (1.9)	9036 (1.9)
Ethnicity			
White	238,441 (94.1)	204,496 (94.1)	442,937 (94.1)
Mixed race	1738 (0.7)	1066 (0.5)	2804 (0.6)
Asian or Asian British	5303 (2.1)	5551 (2.6)	10,854 (2.2)
Black or Black British	4401 (1.7)	3213 (1.5)	7614 (1.6)
Other	2432 (1.0)	1876 (0.9)	4308 (0.9)
Unknown^a^	1029 (0.4)	1225 (0.6)	2254 (0.5)
Smoking status and intensity, cigarettes per day			
Never	151,008 (59.6)	106,404 (50.0)	257,412 (54.7)
Former, <15	16,717 (6.6)	11,565 (5.3)	28,282 (6.0)
Former, ≥15	31,333 (12.4)	42,615 (19.6)	73,948 (15.7)
Former, unknown	30,434 (12.0)	28,416 (13.1)	58,850 (12.5)
Current, <15	8270 (3.4)	6058 (2.8)	14,328 (3.0)
Current, ≥15	8850 (3.5)	10,854 (5.0)	19,704 (4.1)
Current, unknown	5412 (2.1)	10,183 (4.7)	15,595 (3.3)
Unknown^a^	1320 (0.5)	1332 (0.6)	2652 (0.5)
Alcohol consumption, grams per day			
<1	38,298 (15.1)	14,403 (6.6)	52,701 (11.1)
≥1–<10	99,386 (39.2)	47,807 (22)	147,193 (31.2)
≥10–<20	55,341 (21.8)	46,306 (21.3)	101,647 (21.6)
≥20–<30	21,887 (8.6)	36,715 (16.9)	58,602 (12.4)
≥30+	12,637 (5.0)	57,476 (26.4)	70,113 (14.9)
Non-drinkers	23,898 (9.4)	13,573 (6.2)	37,471 (7.9)
Unknown^a^	1904 (0.8)	1151 (0.5)	3055 (0.6)
Physical activity, metabolic equivalents (METS) per week			
Low (0–<10)	46,164 (18.2)	43,264 (19.9)	89,428 (18.9)
Moderate (≥10–<50)	101,089 (39.9)	90,236 (41.5)	191,325 (40.6)
High (≥50)	39,741 (15.7)	43,090 (19.8)	82,831 (17.6)
Unknown^a^	66,320 (26.1)	40,816 (18.7)	10,7136 (22.7)

Results shown are *N* (%) unless otherwise specified.

^a^This category included participants who responded either ‘Do not know’ or ‘prefer not to say’.

**Table 2:** Hazard ratios (HRs) and 95% confidence intervals (CIs) comparing risk of cancer in men to women.

	Women (%)	Men (%)	Total count	Minimally-adjusted model^a^		Multivariable-adjusted model^b^		Multivariable-adjusted model with cancer specific covariates^c^		
				HR (95% CI)	*p*-value	HR (95% CI)	*p*-value	Additional covariates	HR (95% CI)	*p*-value
**Oral cavity [C00-C14]**	324 (33.8)	634 (66.2)	958	2.34 (2.04–2.67)	<0.0001	2.06 (1.69–2.51)	<0.0001		2.06 (1.69–2.51)	<0.0001
**Oesophagus [C15]**	249 (26.0)	707 (74.0)	956	3.30 (2.86–3.82)	<0.0001	2.89 (2.36-3.55)	<0.0001		2.89 (2.36–3.55)	<0.0001
Adenocarcinoma	110 (16.2)	571 (83.8)	681	6.01 (4.90–7.37)	<0.0001	5.47 (4.19–7.14)	<0.0001	Gastro-oesophageal reflux disease	5.45 (4.18–7.12)	<0.0001
Squamous cell carcinoma	128 (52.5)	116 (47.5)	244	1.07 (0.83–1.37)	0.622	0.81 (0.55–1.20)	0.297		0.81 (0.55–1.20)	0.297
**Stomach [C16]**	203 (31.3)	446 (68.7)	649	2.55 (2.16–3.01)	<0.0001	2.34 (1.83–2.97)	<0.0001		2.34 (1.83–2.97)	<0.0001
Gastric cardia [C16.0]	62 (21.8)	223 (78.2)	285	4.17 (3.15–5.53)	<0.0001	3.70 (2.51–5.44)	<0.0001	Diabetes	3.65 (2.48–5.38)	<0.0001
Gastric non-cardia [C16.1–C16.6]	77 (39.9)	116 (60.1)	193	1.74 (1.31–2.33)	0.0016	1.69 (1.09–2.60)	0.018		1.69 (1.09–2.60)	0.018
**Colorectum [C18-C20]**	2316 (43.0)	3072 (57.0)	5388	1.55 (1.47–1.64)	<0.0001	1.25 (1.15–1.36)	<0.0001	Diabetes, inflammatory bowel disease, processed meat	1.22 (1.12–1.33)	<0.0001
Colon [C18]	1667 (46.6)	1909 (53.4)	3576	1.34 (1.25–1.43)	<0.0001	1.06 (0.96–1.17)	0.254	Diabetes, inflammatory bowel disease, processed meat	1.04 (0.94–1.15)	0.456
Rectum [C19–C20]	663 (36)	1178 (64)	1841	2.08 (1.89–2.29)	<0.0001	1.74 (1.51–2.01)	<0.0001	Diabetes, inflammatory bowel disease, processed meat	1.70 (1.47–1.96)	<0.0001
**Anus [C21]**	127 (65.8)	66 (34.2)	193	0.62 (0.46–0.83)	0.001	0.45 (0.29–0.71)	<0.0001	Human immunodeficiency virus (HIV), no. of sexual partners	0.41 (0.26–0.64)	<0.0001
**Liver [C22]**	194 (33.4)	387 (66.6)	581	2.34 (1.97–2.79)	<0.0001	2.00 (1.55–2.58)	<0.0001	Alcoholic liver disease, non-alcoholic fatty liver disease, gallbladder disease	1.91 (1.48–2.47)	<0.0001
**Gallbladder [C23]**	74 (63.8)	42 (36.2)	116	0.64 (0.44–0.94)	0.023	0.56 (0.31–0.99)	0.046	Gallbladder disease	0.55 (0.31–0.97)	0.0036
**Pancreas [C25]**	531 (45.9)	627 (54.1)	1158	1.38 (1.23–1.55)	<0.0001	1.04 (0.87–1.25)	0.642		1.04 (0.87–1.25)	0.642
**Lung [C34]**	1779 (48.6)	1885 (51.4)	3664	1.23 (1.16–1.32)	<0.0001	0.93 (0.84–1.03)	0.144		0.93 (0.84–1.03)	0.144
Adenocarcinoma	825 (54.2)	697 (45.8)	1522	0.98 (0.89–1.09)	0.759	0.72 (0.62–0.84)	<0.0001		0.72 (0.62–0.84)	<0.0001
Squamous cell carcinoma	293 (37.4)	490 (62.6)	783	1.94 (1.67–2.24)	<0.0001	1.41 (1.14–1.75)	0.002		1.41 (1.14–1.75)	0.002
Small cell carcinoma	156 (45)	191 (55)	347	1.42 (1.15–1.75)	0.001	1.19 (0.86–1.64)	0.289		1.19 (0.86–1.64)	0.289
**Melanoma [C43]**^**d**^	1335 (49.3)	1371 (50.7)	2706	1.22 (1.13–1.31)	<0.0001	0.87 (0.77–0.98)	0.019	Use of UV protection, time spent outdoors in the summer, ease of skin tanning	0.97 (0.86–1.09)	0.591
**Breast [C50]**	8737 (99.4)	55 (0.6)	8792	0.007 (0.006–0.009)	<0.0001	0.005 (0.004–0.007)	<0.0001		0.005 (0.004–0.007)	<0.0001
**Kidney [C64-C65]**	492 (35.7)	885 (64.3)	1377	2.11 (1.89–2.36)	<0.0001	1.84 (1.56–2.16)	<0.0001	Hypertension	1.77 (1.51–2.09)	<0.0001
**Bladder [C67]**	237 (22.2)	830 (77.8)	1067	4.07 (3.53–4.71)	<0.0001	3.47 (2.85–4.24)	<0.0001		3.47 (2.85–4.24)	<0.0001
**Thyroid [C73]**	292 (73.7)	104 (26.3)	396	0.43 (0.34–0.53)	<0.0001	0.35 (0.25–0.48)	<0.0001	Goitre	0.36 (0.26–0.49)	<0.0001
**Lymphatic and haematopoietic cancers [C81-C96]**	1886 (43.7)	2431 (56.3)	4317	1.51 (1.42–1.60)	<0.0001	1.22 (1.11–1.33)	<0.0001		1.22 (1.11–1.33)	<0.0001
Hodgkin lymphoma [C81]	63 (47.7)	69 (52.3)	132	1.31 (0.93–1.84)	0.122	1.29 (0.77–2.17)	0.326		1.29 (0.77–2.17)	0.326
Non-Hodgkin lymphoma [C82-C85]	931 (46.4)	1074 (53.6)	2005	1.35 (1.24–1.47)	<0.0001	1.04 (0.91–1.19)	0.585		1.04 (0.91–1.19)	0.585
Multiple myeloma [C90 and C88]	383 (43.1)	505 (56.9)	888	1.54 (1.35–1.76)	<0.0001	1.35 (1.10–1.65)	0.004		1.35 (1.10–1.65)	0.004
Leukaemia [C91–C95]	500 (39.5)	766 (60.5)	1266	1.8 (1.60–2.01)	<0.0001	1.43 (1.21–1.69)	<0.0001		1.43 (1.21–1.69)	<0.0001

^a^Minimally-adjusted hazard ratios (95% CIs) stratifying for age, fifths of Townsend deprivation index and region.

^b^Multivariable-adjusted hazard ratios (95% CIs), additionally adjusted for ethnicity, qualifications, height, BMI, smoking (status and intensity), and alcohol.

^c^Multivariable-adjusted hazard ratios (95% CIs) with additional adjustment for cancer-specific covariates. Covariate selection has been described in the text and in Supplementary tables.

^d^The multivariable-adjusted Cox model for melanoma was run separately in those aged <65 years and ≥65 years, to address violations of the proportional hazard assumption. For participants aged <65 years (including 1177 cases), risk of cancer was lower in men compared to women (HR = 0.73, 95% CI 0.61–0.87: *p* value = 0.001). For those aged ≥65 years (1529 cases), risk of cancer was nominally higher in men compared to women (HR = 1.21, 95% CI 1.03-1.42; *p* value = 0.01).

### Associations between sex and cancer endpoints

We observed significant differences in risk by sex for many cancers; men were at greater risk of cancer at most sites than women (Fig. 1 and Table 2). In the minimally adjusted models, the highest HRs for cancer in men compared to women were for oesophageal adenocarcinoma (HR 6.01; 95% CI 4.90–7.37), followed by cancers of the gastric cardia (HR 4.17; 95% CI 3.15–5.53), and bladder (HR 4.07; 95% CI 3.53–4.71). The lowest HRs in men compared to women were for cancers of the breast (HR 0.007; 95% CI 0.006–0.009), followed by thyroid (HR 0.43; 95% CI 0.34–0.53), and anus (HR 0.62 95% CI 0.46–0.83).

In the multivariable models with additional adjustment for cancer site-specific risk factors, men had a higher risk than women of oesophageal adenocarcinoma (HR 5.45; 95% CI, 4.18–7.12), and of cancers of the gastric cardia (HR 3.65; 95% CI 2.48–5.38), bladder (HR 3.47; 95% CI 2.85–4.24), oral cavity (HR 2.06; 95% CI 1.69–2.51), liver (HR 1.91; 95% CI 1.48–2.47), kidney (HR 1.77; 95% CI 1.51–2.09), rectum (HR 1.70; 95% CI 1.47–1.96), and leukaemia (HR 1.43; 95% CI 1.21–1.69) (Fig. 1 and Table 2). In men compared to women, the lowest risks after breast cancer were observed for cancers of the thyroid (HR 0.36; 95% CI, 0.26–0.49), anus (HR 0.41; 95% CI, 0.26–0.64), and lung adenocarcinoma (HR 0.72; 95% CI 0.62–0.84). For ten cancer sites, the sex differences were attenuated in multivariable-adjusted models and were no longer significant at *p* < 0.0017 (Fig. 1 and Table 2). These were cancers of the colon (HR 1.04; 95% CI 0.94-1.15), lung (HR 0.93; 95% CI 0.84–1.03), lung small cell carcinoma (HR 1.19; 95% CI 0.86–1.64), pancreas (HR 1.04; 95% CI 0.87–1.25), Hodgkin lymphoma (HR 1.29; 95% CI 0.77–2.17), NHL (HR 1.04; 95% CI 0.91–1.19), melanoma (HR 0.97; 95% CI 0.86–1.09), gastric non-cardia (HR 1.69; 95% CI 1.09-2.60), gallbladder (HR 0.55; 95% CI 0.31-0.97), and multiple myeloma (HR 1.35; 95% CI 1.10–1.65). The multivariable-adjusted Cox model for melanoma was run separately in those aged <65 years and ≥65 years, to address violations of the proportional hazards assumption. For participants aged <65 years (including 1177 cases), risk of cancer was lower in men compared to women (HR = 0.73, 95% CI 0.61–0.87: *p*-value = 0.001). For those aged ≥65 years (1529 cases), risk of cancer was nominally higher in men compared to women (HR = 1.21, 95% CI 1.03–1.42; *p*-value = 0.01).

### Sensitivity analyses

We examined sex differences in cancer risk in the subset of never smokers with 257,390 participants of whom 150,996 (58.7%) were women and 106,394 (41.3%) men (Supplementary Table 3). Among them, 14,659 participants were diagnosed with a malignant cancer of interest, of whom 9881 (67.4%) were women and 4778 (32.6%) men. Not all endpoints had enough cases for a robust analysis, however, the HRs were mostly consistent when compared with the main multivariable-adjusted analyses. Cancer of the stomach had a HR attenuated closer to 1 (HR 1.5; 95% CI 1.03–2.2, *p* = 0.037), however there were not enough cancer cases to detect whether this was due to a greater proportion of gastric noncardia cancers in never smokers, or some residual effects of smoking in the multivariable-adjusted models.

We further examined sex differences in cancer risk in the subset of light alcohol drinkers with 301,509 participants, of whom 192,999 (64.0%) were women and 108,510 (36.0%) men (Supplementary Table 4). Among them, 20,286 participants were diagnosed with a malignant cancer of interest, of whom 14,037 (69.2%) were women and 6249 (30.8%) men. After multivariable adjustments, the HRs were consistent with those in the main analyses for most cancer endpoints, except for cancers of the oral cavity (HR 1.54; 95% CI 1.17–2.02, *p* = 0.002), which somewhat attenuated.

Furthermore, restricting analyses to participants with the highest qualification attainment group (Supplementary Table 5), additionally adjusting for height using a quadratic term (Supplementary Table 6), and additionally adjusting for physical activity (Supplementary Table 7) did not materially alter the HRs for any of the cancer endpoints.

## Discussion

In this first comprehensive prospective analysis of sex differences in the risk of cancer performed in the UK we found evidence of marked sex differences in the incidence of several cancers that were not explained by differences in established risk factors. Notably, men compared to women had a higher risk of eight cancers, the largest difference being for oesophageal adenocarcinoma, followed by cancers of the gastric cardia, bladder, oral cavity, liver, kidney, and rectum, and leukaemia. Women compared to men had a higher risk of four cancers, with the highest relative risk for breast cancer, followed by cancers of the thyroid, and anus, and lung adenocarcinoma. Furthermore, for melanoma, the differences in incidence by sex varied by age; under the ages of 65, women had a higher risk compared to men, whereas over the age of 65 men had a higher risk than women. For all the above-mentioned sites, the differences in risk by sex were attenuated a little but remained after further adjustment for established cancer risk factors that may play a role in contributing to the differences.

The findings of our analyses are consistent with the one previous pan-cancer epidemiological study that has investigated sex differences within a prospective cohort study [6] and with studies that have used national-level registries [4, 5, 8, 9, 22–24]. Explanations for sex differences in cancer incidence may include sex differences in prevalence and/or effects of other risk factors and emerging risk factors that may include dietary, molecular, genetic, and environmental factors. Previous research on sex differences in cancer incidence has suggested height (possibly as a proxy for cell number) may explain part of the sex differences [25, 26] and while our analyses reiterated the association of height with cancer risk, accounting for the height differences explained some but not all the sex differences. Moreover, the results from sensitivity analyses restricted to participants who had never smoked and were light drinkers of alcohol suggested that residual confounding by smoking and alcohol, respectively, was unlikely to explain the sex differences in cancer incidence for sites including bladder, kidney, liver, lung adenocarcinoma, oesophageal adenocarcinoma, rectum, and thyroid. Here, we reflect on those cancer endpoints and discuss other potential risk factors that may be contributing to their respective sex differences in incidence and highlight where future research may be valuable.

### Oesophageal adenocarcinoma

The excess risk of oesophageal adenocarcinoma in men, which was more than fivefold greater than women in the current study, has been attributed to a higher prevalence of obesity in men, and of gastro-oesophageal reflux disease, and their relationship with each other [27, 28]. Our study adjusted for both factors and found that neither risk factor explained much of the sex difference. However, since BMI does not capture fat distribution entirely and there are substantial sex differences in fat distribution, other potential explanations may include the role of higher abdominal obesity in men, which contributes to increased chronic gastric pressure and increased cancer risk [28–30]. Furthermore, oestrogen has been proposed to confer a protective effect in women [29, 31].

### Bladder

The high incidence of bladder cancer in men compared to women (more than threefold in the UK Biobank) is not fully understood but may be due to a combination of differences in carcinogenic exposures, hormones, and genetic differences by sex [32]. Men are also more likely to have higher carcinogenic exposures through occupational exposures [33–35]. These include but are not limited to occupations related to aluminium production, rubber production, painting, and firefighting [33–35]. Data on hormones suggest that oestrogens may be protective for bladder cancer in women, but the role of androgens is unclear [33, 36]. Furthermore, genetic studies suggest the mosaic loss of Y chromosome in blood in men may be associated with increased bladder cancer risk in men and some X chromosome-linked genes (*KDM6A*) may confer protection in women [37, 38]. Men and women generally have an equal expression of X-linked genes, as during embryogenesis, one of the two X chromosomes in women randomly undergoes inactivation and there is haploid expression of the X chromosome in each cell [38]. However, some genes (termed EXITS genes, derived from ‘escape from X-inactivation tumour suppressors’) may escape the inactivation process rendering them to have biallelic expression in women. This group of genes includes tumour suppressor genes (including *KDM6A*) and thus may confer protection in women as inactivation would require two deleterious mutations whereas for men it would require one [38]. It has also been hypothesised that bladder cancer symptoms in women may also be mistaken for more common conditions such as urinary tract infections, for which they may be treated with several antibiotic courses first before undergoing urological investigations [32]. However, these delays may only have a modest impact [39] and it is unlikely that they contribute materially to the observed sex differences in incidence.

### Liver

We observed a nearly twofold higher risk of cancer of the liver in men compared to women, even after accounting for alcohol related liver diseases and restricting the analyses to participants with low alcohol consumption. While the possibility of residual confounding by alcohol consumption may remain, this is unlikely to entirely explain the sex difference in liver cancer risk. To some extent, the excess risk in men may be attributed to infections [40], as there are sex differences in the seroprevalence of hepatitis B infections in the UK. According to the UK Health Security Agency, the estimated prevalence of hepatitis B surface antigen in the population in 2022 was 0.41% (95% CI 0.36-0.46) in women and 0.77% (95% CI 0.64-0.92) in men [41]. Additionally, there are sex differences in the immunological responses mounted to chronic hepatitis infections, which may further contribute to the disparity [42]. A prospective cohort study in the UKB also suggested sex-specific associations of metabolic syndrome and its components with liver cancer, with central obesity and hyperglycaemia associated with elevated risk of cancer in men but not in women [43].

### Kidney

Similar to the results of our analysis, a previous cross-sectional study [44] reported the relative risk of kidney cancer was twice as high in men compared to women, a difference that was consistent by age, year of diagnosis, and region worldwide. They hypothesised that some of this sex difference may be attributed to sex differences in smoking, BMI, and hypertension, which our analyses accounted for and yet we still observed a 1.7-fold increase in risk of kidney cancer in men. Other potential risk factors may include occupational hazards, including but not limited to working with herbicides and factory agents (associated with an increased risk of cancer in men) [45], reproductive and hormonal risk factors [46], and genetic differences [47, 48]. A genome-wide association study (GWAS) [48] investigating sex differences of RCC reported sex-specific associations for two RCC risk SNPs (*DPF3* at 14q24 expressed in women more than men, and *EPAS1* at 2p21 expressed in men more than women), which advocates the need for more well-powered genetic studies to further elucidate these sex differences.

### Rectum

As has been observed in other studies [6, 49], in UKB we observe a higher risk of cancer of the rectum (1.7-fold) in men compared to women, but not of colon cancer, even after accounting for colorectal cancer risk factors. Potential explanations for this sex difference in rectal cancer incidence may include sex hormone-related mechanisms, with some evidence of oestrogen and progesterone conferring a protective effect in women [50]. Although not well powered due to the rarity of the syndromes, studies reported that men with Klinefelter syndrome (an extra copy of the X chromosome) have a reduced risk of colorectal cancer and women with Turner syndrome (only one copy of the X chromosome) have an increased risk [50]. Sex differences in visceral adiposity traits may also explain some risk differences as studies have reported interactions with obesity traits and sex in relation to colorectal cancer risk [30]. Differences in screening uptake have been suggested to be another contributory factor, as men generally have lower rates of screening uptake (both colonoscopies and sigmoidoscopies) [49]. However, if screening differences explained some excess risk for rectal cancer, we might expect to observe a somewhat similar sex differences for colon cancer. Further research is needed to explore risk factors specific to rectal cancer development in men that may explain their excess risk.

### Lung adenocarcinoma

The overall lung cancer risk was higher in men compared to women in minimally adjusted models, but this excess risk was eliminated after adjusting for smoking and other factors. The higher risk of lung adenocarcinoma in women compared to men that we observed in the UKB has been consistently reported in other epidemiological studies, however, conclusive evidence on risk factors that may explain the excess risk is limited [51–53]. Possible explanations include sex differences in chronic lung conditions such as asthma, which has a higher prevalence in women in adulthood and may be linked with increasing cancer risk with persistent damage through inflammation or trauma [54]. Our sensitivity analysis in never smokers adjusted for exposure to tobacco smoke at home and outside home in participants who identified as never smokers, however, the increased risk in women remained significant. Cross-sectional studies on molecular signatures in tumour tissue in The Cancer Genome Atlas have suggested marked sex differences in molecular profiles of patients with lung adenocarcinoma [55]. For example, somatic mutations of the *STK11* gene were more frequent in men compared to women, whereas mutations in the *DMD* gene were more frequent in women [55]. More higher-powered studies in the genetics of lung adenocarcinoma are needed to better understand the sex differences.

### Thyroid

We observed the risk of thyroid cancer was almost 2.7-fold higher in women compared to men; an excess risk that has been investigated in other epidemiological studies [56, 57] previously but suggested to be not a real sex difference in incidence and instead an artefact of diagnostic differences between men and women. A cross-sectional analysis of US adults (using the National Cancer Institute’s Surveillance, Epidemiology, and End Results Programme data) reported the sex disparity in thyroid cancer was confined to small subclinical papillary thyroid cancers and they were identified more commonly in women than men, with its subclinical prevalence being similar (1:1) between the sexes and with men being diagnosed later in life [57]. They suggest it could be due to women’s behaviour towards accessing healthcare services more commonly than men and being referred for thyroid ultrasonography for general concerns including fatigue and menstrual disturbances [56, 57]. It is also hypothesised that hormonal and reproductive factors (pregnancy, parity, number of live births, contraceptive pill, and menopausal status) in women may contribute to excess risk, however, a meta-analysis of results from studies of these risk factors did not find consistent associations with increased risk [58].

### Strengths and limitations

Notable strengths of the analyses include them being performed in one of the largest prospective cohort studies to date, being well powered for most cancer sites, with most relevant risk factors associated with respective cancers measured in the cohort, and a literature-based approach to identifying those risk factors. The study design also allowed investigation of the most common cancer sites, thus avoiding outcome selection bias and maintaining consistency in risk estimation.

Nonetheless, there are some limitations; first, it is likely that our analyses are subject to some residual confounding, including, for example imperfectly measured smoking and alcohol consumption. This is notably relevant for cancers of the anus, gastric cardia, and oral cavity, and for leukaemia, where relative risks in men compared to women were somewhat attenuated in analyses restricted to never smokers or light drinkers (or for some cancers could not be analysed as the subgroups did not have enough cases for a robust analysis). There were putative or established risk factors that were not included in our models either because they were not captured in the cohort, or because they were traits with some but limited evidence of their associations with cancer sites. These included occupations, carcinogenic infections, comorbidities, and traits (for example, metabolic syndrome, body composition measures). Further research is warranted to assess the extent to which residual confounding might explain the apparent sex difference for these cancers. Second, the generalisability of our study was limited as the UKB cohort study comprises mostly white European participants and participants who are relatively healthier than the UK population, and it would be useful to replicate a similar analysis in other study populations. Furthermore, in UK Biobank, it is not currently possible to distinguish sex from gender due to the survey questions, but this represents an important avenue for future research in prospective cohorts where this distinction can be factored into analyses. Third, it may also be useful to replicate these analyses in prospective cohort studies with more cases for some cancer endpoints, including cancers of the gastric cardia, anus, and gallbladder, which had statistically significant or nearly statistically significant increased risk for one sex but could not be explored further due to relatively low power. Fourth, we did not explore sex differences in risk for some cancers by anatomical subsites, nor did we have information on tumour stage or histological grade, and analyses stratified by these categories may be insightful.

### Concluding remarks

In our pan-cancer analyses in a large UK cohort study, we have identified sex differences in cancer incidence that are only partially explained by known cancer risk factors. Particularly marked differences include a higher incidence of oesophageal adenocarcinoma and of cancers of the bladder, rectum, liver, and kidney in men, and of thyroid and lung adenocarcinoma in women. Future research on the unexplained sex differences in cancer risk between men and women may provide insights into novel aetiological pathways for cancer.

## Supplementary information

Supplementary Information

## Data Availability

Researchers can apply to use the UK Biobank resource for health-related research that is in the public interest (https://www.ukbiobank.ac.uk/register-apply/).
