# Supplementary material for: Sex differences in cancer incidence: prospective analyses in the UK Biobank
Source: Br J Cancer. 2025 May 8;133(2):216–26. doi: 10.1038/s41416-025-03028-y (PMC12304391; doi:10.1038/s41416-025-03028-y)
Supplement: Supplementary file 1 — Supplementary Information [file 41416_2025_3028_MOESM1_ESM.pdf]

Supplementary Information

Sex differences in cancer incidence: prospective analyses in the UK Biobank

Maira Khan[1], Keren Papier[1], Kirstin L. Pirie[1], Tim J. Key[1], Joshua Atkins[1] and Ruth C. Travis[1]

[1] Cancer Epidemiology Unit, Nuffield Department of Population Health, University of Oxford, Oxford, UK

Correspondence to: Maira Khan, Cancer Epidemiology Unit, Nuffield Department of Population Health, University of Oxford, Richard Doll Building, Roosevelt Drive, Oxford OX3 7LF, UK.

Tel: +44 1865 289600

Fax: +44 1865 289610

Email: maira.khan@ndph.ox.ac.uk | maira.khan@lmh.ox.ac.uk

ORCID ID: https://orcid.org/0009-0003-2524-5856

Table of Contents

Abbreviations 3
Methods 4
Data collection and categorization of covariates 4
Age at recruitment 4
Region 4
Townsend Deprivation Index 4
BMI 4
Height 4
Ethnicity 4
Qualifications 5
Smoking 5
Alcohol 6
Physical Activity 6
Prevalent Diseases 6
.... 8
Supplementary Figure 1: UK Biobank cohort exclusion criteria 8
Supplementary Table 1: Histology codes for distinguishing cancer subtypes 9
Supplementary Table 2: Prevalent diseases definitions 11
Supplementary Table 3: Cancer specific multivariable-adjusted Cox proportional hazards models comparing risk of cancer incidence in men versus women who identified as never smokers at recruitment in the UK Biobank. 13
Supplementary Table 4: Cancer specific multivariable-adjusted Cox proportional hazards models comparing risk of cancer incidence in men versus women who identified as light alcohol drinkers (<20 g/day) in the UK Biobank 15
Supplementary Table 5: Cancer specific multivariable-adjusted Cox proportional hazards models comparing risk of cancer incidence in men versus women in the highest qualification attainment stratum (Prof Q/NVQ/HND/HNC/Degree or other professional qualifications) in the UK Biobank. 17
Supplementary Table 6: Cox proportional hazards models comparing risk of cancer incidence in men versus women additional adjustment for non-linear height (height^2) added to the model. 19
Supplementary Table 7: Cox proportional hazards model comparing risk of cancer incidence in men versus women with additional adjustment for physical activity (METS/week)*. 20
Additional references 21

Abbreviations

BMI	Body mass index
CI	Confidence interval
GWAS	Genome-wide association study
HIV	Human immunodeficiency virus
HPV	Human papillomavirus
HR	Hazard ratio
ICD	International Classification of Diseases
MET	Metabolic equivalent
NHS	National Health Service
OPCS-4	Operating procedure codes supplement (version 4)
RCC	Renal cell carcinoma
SCC	Squamous cell carcinoma
UKB	UK Biobank
UV	Ultraviolet

Methods

Data collection and categorization of covariates

Age at recruitment

Age at recruitment (data field 21022) is a derived variable based on a participant's date of birth and date of attending an initial assessment at the recruitment centres. We categorized age into 6 groups, (< 45, 45 to <50, 50 to <55, 55 to <60, 60 to <65, and ≥65 years).

Region

Region refers to the respective baseline assessment centres, which were based in Barts, Hounslow, Croydon, Swansea, Wrexham, Cardiff, Stockport, Manchester, Liverpool, Bury, Newcastle upon Tyne, Middlesbrough, Leeds, Sheffield, Stoke, Birmingham, Nottingham, Oxford, Reading, Bristol, Glasgow, and Edinburgh. They were grouped into 10 regions; namely London (Barts, Hounslow, and Croydon), Wales (Wrexham, Swansea, and Cardiff), North-West (Stockport, Manchester, Liverpool, and Bury), North-East (Newcastle and Middlesbrough), Yorkshire and Humber (Leeds and Sheffield), West Midlands (Stoke and Birmingham), East Midlands (Nottingham), South-East (Oxford and Reading), South-West (Bristol), and Scotland (Glasgow and Edinburgh).

Townsend Deprivation Index

Material deprivation is measured by the Townsend Deprivation Index (TDI). It is a score that incorporates census area data for employment, car ownership, home ownership, and household overcrowding[1]. TDI was derived prior to recruitment using preceding national census output area (data field 22189), and participants were assigned a score corresponding to the output area in which their postcode was located. For the analysis, TDI was categorised using quintiles, and participants without TDI information were categorized as 'unknown' (0.1%).

BMI

Body mass index (data field 23104) was calculated as weight in kilograms divided by height in meters squared. Participant weight was measured at baseline using a Tanita BC418MA body composition analyser as part of Bioimpedance measurement. For participants that did not participate in a Bioimpedance analysis, body weight was based on measurement using a standard scale. Where height and/or weight measurements were missing, participants were categorized into an 'unknown group' and dropped from the analytical cohort (0.6%).

Height

Standing height (data field 50) was measured at baseline assessment with a Seca 240 cm height measure using a standardized protocol. Where height measurements were missing participants were categorized into an 'unknown' group (0.5%).

Ethnicity

Ethnicity was assessed using one question on ethnicity 'What is your ethnic group?' (data field 21000) with options for 'White', 'Mixed', 'Asian or Asian British', 'Black or Black British', 'Chinese', 'Other ethnic group', 'Do not know' or 'Prefer not to answer' and four supporting questions within each

group. ‘White’ (data field 1667) was defined as either answering white, British, Irish or any other white background; ‘Asian or Asian British’ was defined as Asian or Asian British, Chinese, Indian, Pakistani, Bangladeshi, or any other Asian background; ‘Black or Black British’ (data field 3690) was defined as Black or Black British, Caribbean, African or any other Black background; ‘Mixed Race or other’ (data field 3819) was defined as reporting either any other ethnic groups or mixed ethnicity; participants with missing data, reporting they did not know or preferred not to answer, were grouped as ‘unknown’ (0.6%).

Qualifications

Highest qualification was assessed within the touchscreen questionnaire ‘Which of the following qualifications do you have?’ (data field 6138), with the options ‘College or university degree’, ‘A levels/AS levels or equivalent’, ‘O levels/GCSEs or equivalent’, ‘CSEs or equivalent’, ‘NVQ or HND or HNC or equivalent’, ‘Other professional qualifications e.g: nursing, teaching’, ‘None of the above’ and ‘Prefer not to answer’. Qualification was then categorized into three groups: the highest qualifications included ‘College or university degree’, ‘NVQ or HND or HNC or equivalent’, ‘Other professional qualifications e.g.: nursing, teaching’ and are referred to as college or university degree/vocational qualification; the intermediate qualifications included ‘A levels/AS levels or equivalent’ and are referred to as national examination at age 17-18; and the lowest level of qualifications included ‘O levels/GCSEs or equivalent’, ‘CSEs or equivalent’ and are referred to as national examination at age 16. Participants who had answered ‘None of the above’ or ‘prefer not to answer’ or who had not answered the question were categorized as ‘unknown/other’ (18.6%).

Smoking

Smoking status (data field 20116) was derived from two touchscreen questionnaires at baseline, ‘Do you smoke tobacco now?’ (field 1239) with four options, ‘Yes, on most or all days’, ‘Only occasionally’, ‘No’ and ‘Prefer not to answer’, and ‘In the past, how often have you smoked tobacco?’ (field 1249) with the options ‘Smoked on most or all days’, ‘Smoked occasionally’, ‘Just tried once or twice’, ‘I have never smoked’, and ‘Prefer not to answer’. Number of cigarettes per day was obtained from the question ‘About how many cigarettes do you smoke on average each day’ (data field 3456), and participants could either give a number or answer, ‘less than one’, ‘do not know’ and prefer not to answer’. We created a composite variable combining smoking status and smoking intensity, categorising it into 7 groups of ‘never smokers’, ‘former smokers <15 cigarettes/day’, ‘former smokers >=15 cigarettes/day’, ‘current smokers <15 cigarettes/day’, ‘current smokers >=15 cigarettes/day’, ‘current smokers, amount unknown’, and an ‘unknown’ smoking status (0.6%). Exposure to second hand smoke was measured using two variables (data field 1269 and 1279), and only in participants who self-reported as never smokers (n=257,412). They were asked ‘At your home, how many hours per week are you exposed to other people’s tobacco smoke? (participants could select from 0 to 168 hours, do not know, or prefer not to answer), and ‘Outside of your home, how many hours per week are you exposed to other people’s tobacco smoke? (with options ranging from 0-168 hours, do not know, and prefer not to answer). 18,970 participants who identified as never smokers reported they were exposed to at least 1 or more hours per week of tobacco smoke at home, with 61.1% women in this category. 77,847 participants who identified as never smokers reported they were exposed to at least 1 or more hours per week of tobacco smoke outside home, with 46.1% women in this category. The multivariable-adjusted model for lung adenocarcinoma adjusted for exposure to tobacco smoke at home and outside home in a continuous format.

Alcohol

Alcohol drinker status (data field 20117) was derived at recruitment and participants were categorised as current drinkers, previous drinkers, never drinkers, and those who preferred not to answer. One question on alcohol intake frequency (data field 1558) was 'about how often do you drink alcohol?' categorised into 'daily or almost daily', 'three to four times per week', 'once or twice a week', 'one to three times a month', 'special occasions only', 'never', and 'prefer not to answer'. Further alcohol intake was assessed from 12 questions that participants were asked about their monthly and weekly intake (6 each) for different categories of alcohol. They included questions about red wine (data field 1568 and 4407) 'in an average week/month, how many glasses of red would you drink? (there are six glasses in an average bottle)', white wine or champagne (data fields 1578 and 4418) 'in an average week/month, how many glasses of white wine or champagne would you drink? (there are six glasses in an average bottle)', beer plus cider (data fields 1588 and 4429) 'in an average week/month, how many pints of beer or cider would you drink? (include bitter, lager, stout, ale, Guinness)', spirits (data fields 1598 and 4440) 'in an average week/week, how many measures of spirits or liqueurs would you drink? (there are 25 standard measures in a normal sized bottle; spirits include drinks such as whisky, gin, rum, vodka brandy)' and fortified wine (data fields 1608 and 4451) 'in an average week/month how many glasses of fortified wine would you drink? (there are 12 glasses in an average bottle) (fortified wines include drinks such as sherry, port, vermouth)'. Red wine, white wine, spirits, and fortified wines were assigned an average of 10g alcohol per serving; beer, and cider 20g alcohol per serving. Total drinking per day was calculated from total alcohol intake per week and month, where weekly intakes were unavailable. Total drinking was categorised into four groups: 'never-drinkers', '<1g/day', '1-<10g/day', '10-<20g/day', '≥20g/day', and 'unknown' (0.7%). Participants that reported their overall drinking frequency was drinking only at special occasions were added to the group of those drinking less than 1g/day and those reporting never drinking alcohol were categorised as never-drinkers.

Physical Activity

Physical activity was assessed using a list of questions within the baseline touchscreen questionnaire (data category 54) that were adapted from the short form International Physical Activity Questionnaire (IPAQ)[2]. The questionnaire assesses frequency, duration and of three types of activity (walking (data field 864), moderate activity (data field 884), and vigorous activity (data field 904)). In the UK Biobank cohort, the question on durations was only asked if the participants answered the question on frequency with a number other than zero. Total physical activity was then computed as the sum of walking (2.3 excess metabolic equivalents (METS)), moderate activity (3.0 excess METS), and vigorous activity (7.0 METS) (for at least 10 minutes continuously). If an activity category was missing it was treated as zero metabolic equivalents. Excess METs were categorised into low (<10 METS/week), moderate (10-<50 METS/week), and high (≥50 METS/week) groups. Excess METS represent the energy levels above the basal metabolic resting rates. These cut-offs were obtained with reference from two other papers that had classified physical activity levels in the UK Biobank ([3,4]).

Prevalent Diseases

Supplementary Table 2 highlights the ICD-10, ICD-9, and/or OSPC-4 codes that were used to identify prevalent diseases that were added to the multivariable-adjusted models. All conditions were identified from a maximal sample analysis of 470,771 participants. Furthermore, participants also self-reported diseases during the interview portion of their assessment conducted by a medical professional at the recruitment centre on their past and current medical conditions (data-field 20002).

The codes relevant for the diseases of interest to our study have been further listed in Supplementary Table 2.

Supplementary Figure 1: UK Biobank cohort exclusion criteria

502,386 participants contributed to the UK Biobank cohort

↓

Participants excluded from the cohort analyses if data were missing on:

Participant ID, sex (self reported), region, age at recruitment
Standing height and BMI = 3105

Data on 499,142 participants available

↓

Additional exclusions:

28,143 participants with prevalent cancers (excluding all cancers except non-melanoma skin cancer with international classification of disease code C44)
367 participants with mismatches between self reported sex and genotyped sex

Data on 470,771 participants available*

** For cancer-specific multivariable adjusted models we further excluded participants with an operational history of complete or partial gastrectomy (51 participants). The final cohort size for the cancer specific multivariable adjusted analyses had 470,720 participants. Furthermore, additional exclusions were applied to this cohort size for performing sensitivity analyses.*

Supplementary Table 1: Histology codes for distinguishing cancer subtypes

**WHO ICD-O-3 Oesophageal cancer [ICD C15]**	
**Oesophageal adenocarcinoma**	8140/3 Adenocarcinoma NOS<br>8144/3 Adenocarcinoma, intestinal type<br>8145/3 Adenocarcinoma, diffuse type<br>8246/3 Neuroendocrine carcinoma, NOS<br>8260/3 Papillary, NOS<br>8310/3 Clear cell adenocarcinoma, NOS<br>8312/3 Adenocarcinoma, renal cell type,<br>8323/3 Mixed cell adenocarcinoma<br>8402/3 Nodular hidradenoma, malignant<br>8440/3 Cystadenocarcinoma, NOS<br>8461/3 Serous surface papillary carcinoma<br>8480/3 Mucinous adenocarcinoma<br>8481/3 Mucin-producing adenocarcinoma<br>8490/3 Signet ring cell adenocarcinoma<br>8500/3 Infiltrating duct adenocarcinoma<br>8543/3 Intraductal carcinoma<br>8560/3 Adenocarcinoma and epidermoid carcinoma, mixed
**Oesophageal squamous cell carcinoma**	8070/3 Epidermoid NOS<br>8071/3 Squamous cell carcinoma keratinizing, NOS<br>8081/3 Intraepidermal squamous cell carcinoma
**WHO ICD-O-3 Lung cancer [ICD C34] histological subtypes**<br>**These histology codes had no lung cancer cases.*	
**Lung adenocarcinoma**	8140/3 Adenocarcinoma, NOS<br>8140/6 NOS, metastatic<br>*8190/3 trabecular adenocarcinoma*<br>*8211/3 tubular adenocarcinoma*<br>8244/3 carcinoid and adenocarcinoma<br>8250/3 bronchiolar (C34.1), bronchiolo-alveolar, NOS<br>8251/3 alveolar adenocarcinoma<br>*8252/3 Bronchiolo-alveolar carcinoma, non-mucinous*<br>*8253/3 Bronchio-alveolar goblet cell type adenocarcinoma*<br>*8254/3 Bronchio-alveolar adenocarcinoma: clara cell and goblet cell type, mixed mucinous and non-mucinous, type II pneumocyte and goblet cell type*<br>8255/3 adenocarcinoma combined with other types of carcinomas<br>8260/3 Papillary adenocarcinoma, NOS<br>*8290/3 Oxyphilic adenocarcinoma*<br>8310/3 clear cell adenocarcinoma, NOS<br>8323/3 Mixed cell adenocarcinoma<br>8440/3 Cystadenocarcinoma, NOS<br>8480/3 mucinous adenocarcinoma, NOS<br>8481/3 mucin-producing, mucin secreting<br>8490/3 Signet ring cell adenocarcinoma<br>8550/3 acinar adenocarcinoma<br>*8570/3 adenocarcinoma with squamous metaplasia*<br>*8571/3 adenocarcinoma with cartilaginous and osseous metaplasia*<br>*8572/3 adenocarcinoma with spindle cell metaplasia*<br>*8573/3 adenocarcinoma with apocrine metaplasia*<br>8574/3 adenocarcinoma with neuroendocrine differentiation

	8576/3 Hepatoid adenocarcinoma
**Lung small cell carcinoma**	8041/3 small cell carcinoma, NOS<br>8042/3 oat cell carcinoma<br>8045/3 combined small cell carcinoma (combined small cell-adenocarcinoma, combined small cell-large cell carcinoma, combined small cell-squamous cell carcinoma
**Lung squamous cell carcinoma**	*8051/3 verrucous squamous cell carcinoma*<br>*8052/3 papillary squamous cell carcinoma*<br>8070/3 squamous cell carcinoma, NOS<br>8071/3 squamous cell carcinoma, keratinizing, NOS<br>8072/3 squamous cell carcinoma, large cell, nonkeratinizing, NOS<br>*8073/3 squamous cell carcinoma, small cell, nonkeratinizing*<br>8074/3 squamous cell carcinoma, spindle cell<br>*8075/3 squamous cell carcinoma, adenoid*<br>*8076/3 squamous cell carcinoma, microinvasive*<br>8083/3 basaloid squamous cell carcinoma<br>8084/3 squamous cell carcinoma, clear cell type

References used for the classification along with the ICD-O-3 codes

1. World Health Organization (2013) International Classification of diseases for Oncology, 3rd Edition: First Revision. https://apps.who.int/iris/bitstream/handle/10665/96612/9789241548496_eng.pdf.
2. Ruhl JL, Callaghan C, Schussler N (eds.) Summary Stage 2018: Codes and Coding Instructions, National Cancer Institute, Bethesda, MD, 2022.
3. Watts EL, Perez-Cornago A, Knuppel A, Tsilidis KK, Key TJ, Travis RC, Prospective analyses of testosterone and sex hormone-binding globulin with the risk of 19 types of cancer in men and postmenopausal women in the UK Biobank. *Int. J. Cancer*. 2021; 149: 573–584. https://doi.org/10.1002/ijc.33555
4. Pirie, K., Peto, R., Green, J., Reeves, G.K., Beral, V. (2016), Lung cancer in never smokers in the UK Million Women Study. Int. J. Cancer, 139: 347-354. https://doi.org/10.1002/ijc.30084
5. Jason Y Y Wong, ScD, Bryan A Bassig, PhD, Erikka Loftfield, PhD, Wei Hu, PhD, Neal D Freedman, PhD, Bu-tian Ji, PhD, Paul Elliott, PhD, Debra T Silverman, ScD, Stephen J Chanock, MD, Nathaniel Rothman, MD, Qing Lan, MD, PhD, White Blood Cell Count and Risk of Incident Lung Cancer in the UK Biobank, *JNCI Cancer Spectrum*, Volume 4, Issue 2, April 2020, pkz102, https://doi.org/10.1093/jncics/pkz102

Supplementary Table 2: Prevalent diseases definitions

Prevalent disease/admissions cause	Outcome definition using ICD-10[a]	Outcome definition using ICD-9	Relevant procedure code using OPSC-4 definitions[a]	Touchscreen criteria[b]	Medication criteria[c]	Interview codes for prevalent diseases[d]	Total cases [in women]
Alcoholic Liver Disease	K70					1604	498 [103]
Diabetes Mellitus	E10, E11, E12, E13, E14	250		2443	6177	1220 1222 1223	25,156 [9,760]
Gallbladder disease	K80, K81, K82.9					1161	9,773 [7,327]
Gastrectomy (partial or complete)[e]			G271 G272 G273 G274 G275 G276 G277 G278 G279 G281 G282 G283 G284 G285 G286 G287 G288 G289				51
Gastro-oesophagus reflux disease	K21	530.11 530.81					13,925 [7,037]
Goiter	E049						584 [452]
Human Immunodeficiency Virus	B20 B21 B22 B23 B24	042				1439	455 [<100]
Hypertension	I10					1065	126,410 [59,481]
Inflammatory Bowel Disease	K50 K51 K52.3	555 556				1462 1663 1461	4,580 [2,668]
Non-alcoholic fatty liver disease	K75.8 K76 K74.6						1,174 [561]

[a] Based on OPSC-4 definition and UKB variable 20004.
[b] Used for identifying prevalent diabetes (data-field 2443); participants were asked at recruitment 'Has a doctor ever told you that you have diabetes? (yes/no/do not know/prefer not to answer). Participants that selected 'yes' were classed as those having diabetes.
[c] Used for identifying prevalent diabetes (data field 6177); participants were asked at recruitment 'Do you regularly take any of the following medications? (you can select more than one answer) (cholesterol lowering medication/blood pressure medication/insulin/none of the above/do not know). Participants that selected 'insulin' were classed as those having diabetes.
[d] Based on ICD-10 definition and UKB variable 20002. See text under prevalent diseases.
[e] Participants with complete or partial gastrectomy were excluded for the cancer-specific multivariable adjusted analyses.

Supplementary Table 3: Cancer specific multivariable-adjusted Cox proportional hazards models comparing risk of cancer incidence in men versus women who identified as never smokers at recruitment in the UK Biobank.

	Women (%)	Men (%)	Total count	**HR (95% CI)**	P value	Main analysis results*
Oral cavity [C00-C14]	138 (42.6)	186 (57.4)	324	**1.79 (1.29-2.5)**	5.57e-04	2.06 (1.69-2.51)
Oesophagus [C15]	117 (37.7)	193 (62.3)	310	**2.61 (1.85-3.68)**	4.87e-08	2.89 (2.36-3.55)
Oesophageal adenocarcinoma [C15]	48 (23.6)	155 (76.4)	203	**5.11 (3.25-8.02)**	1.49e-12	5.45 (4.18-7.12)
Oesophageal squamous cell carcinoma [C15]	66 (71.7)	26 (28.3)	92	[insufficient data]	[insufficient data]	0.81 (0.55-1.2)
Stomach [C16]	110 (44)	140 (56)	250	**1.5 (1.03-2.2)**	0.037	2.34 (1.83-2.97)
Gastric cardia [C16.0]	25 (29.4)	60 (70.6)	85	[insufficient data]	[insufficient data]	3.65 (2.48-5.38)
Gastric non-cardia [C16.1-C16.6]	45 (53.6)	39 (46.4)	84	[insufficient data]	[insufficient data]	1.69 (1.09-2.6)
Colorectum [C18-C20]	1266 (50.5)	1242 (49.5)	2508	**1.24 (1.1-1.4)**	3.862e-25	1.22 (1.12-1.33)
Colon [C18]	936 (54.9)	768 (45.1)	1704	**1.01 (0.87-1.17)**	0.875	1.04 (0.94-1.15)
Rectum [C19-C20]	337 (41.5)	476 (58.5)	813	**1.91 (1.54-2.36)**	2.78e-09	1.7 (1.47-1.96)
Anus [C21]	55 (70.5)	23 (29.5)	78	[insufficient data]	[insufficient data]	0.41 (0.26-0.64)
Liver [C22]	92 (43.6)	119 (56.4)	211	**2.39 (1.58-3.62)**	3.67e-05	1.91 (1.48-2.47)
Gallbladder [C23]	31 (68.9)	14 (31.1)	45	[insufficient data]	[insufficient data]	0.55 (0.31-0.97)
Pancreas [C25]	274 (52.6)	247 (47.4)	521	**1.13 (0.87-1.47)**	0.369	1.04 (0.87-1.25)
Lung [C34]	316 (65.7)	165 (34.3)	481	**0.71 (0.53-0.94)**	0.017	0.93 (0.84-1.03)
Lung adenocarcinoma [C34]	174 (68.5)	80 (31.5)	254	**0.62 (0.42-0.93)**	0.002	0.72 (0.62-0.84)
Lung squamous cell carcinoma [C34]	31 (64.6)	17 (35.4)	48	[insufficient data]	[insufficient data]	1.41 (1.14-1.75)
Lung small cell carcinoma [C34]	5 (62.5)	3 (37.5)	8	[insufficient data]	[insufficient data]	1.19 (0.86-1.64)
Melanoma [C43]	823 (53.7)	710 (46.3)	1533	**0.88 (0.75-1.03)**	0.103	0.97 (0.86-1.09)
Breast [C50]	5029 (99.5)	24 (0.5)	5053	**0.005 (0.003-0.007)**	0	0.005 (0.004-0.007)
Kidney [C64-65]	252 (42.7)	338 (57.3)	590	**2.1 (1.64-2.69)**	3.65e-09	1.77 (1.51-2.09)
Bladder [C67]	104 (31.1)	230 (68.9)	334	**3.15 (2.25-4.41)**	1.98e-11	3.47 (2.85-4.24)

	Women (%)	Men (%)	Total count	**HR (95% CI)**	P value	Main analysis results*
Thyroid [C73]	191 (79.6)	49 (20.4)	240	**0.35 (0.23-0.54)**	2.08e-06	0.36 (0.26-0.49)
Lymphatic and haematopoietic [C81-C96]	1083 (49.7)	1098 (50.3)	2181	**1.15 (1.01-1.31)**	0.029	1.22 (1.11-1.33)
Hodgkin lymphoma [C81]	34 (52.3)	31 (47.7)	65	[insufficient data]	[insufficient data]	1.29 (0.77-2.17)
Non-Hodgkin lymphoma [C82-C85]	547 (52.7)	491 (47.3)	1038	**0.92 (0.76-1.11)**	0.387	1.04 (0.91-1.19)
Multiple myeloma [C90 & C88]	221 (47)	249 (53)	470	**1.36 (1.03-1.79)**	0.028	1.35 (1.10-1.65)
Leukaemia [C91-C95]	274 (46.2)	319 (53.8)	593	**1.44 (1.13-1.85)**	0.003	1.43 (1.21-1.69)

Note: This sensitivity analysis was performed on a multivariable-adjusted model that included cancer-specific, and baseline set of covariates. Covariate selection has been described in text and supplementary tables. Cancer endpoints were excluded from the analysis if total counts were less 100. **257,390** participants self-reported as never smokers, of which 14,659 were diagnosed with an incident malignant cancer of interest, with 9,881 cases (67.4%) in women.

*The main analysis results for all participants (HRs and 95% CIs from multivariable-adjusted models with cancer specific covariates) are also shown for comparison.

Supplementary Table 4: Cancer specific multivariable-adjusted Cox proportional hazards models comparing risk of cancer incidence in men versus women who identified as light alcohol drinkers (<20 g/day) in the UK Biobank

	Women (%)	Men (%)	Total count	**HR (95% CI)**	P value	Main analysis results*
Oral cavity [C00-C14]	236 (51.3)	224 (48.7)	460	**1.54 (1.17-2.02)**	0.002	2.06 (1.69-2.51)
Oesophagus [C15]	164 (34.9)	306 (65.1)	470	**2.89 (2.2-3.8)**	3.90e-14	2.89 (2.36-3.55)
Oesophageal adenocarcinoma [C15]	81 (23.1)	269 (76.9)	350	**5.02 (3.58-7.05)**	8.78e-21	5.45 (4.18-7.12)
Oesophageal squamous cell carcinoma [C15]	75 (68.8)	34 (31.2)	109	**0.65 (0.36-1.17)**	0.151	0.81 (0.55-1.2)
Stomach [C16]	148 (42.8)	198 (57.2)	346	**2.30 (1.68-3.15)**	2.23e-07	2.34 (1.83-2.97)
Gastric cardia [C16.0]	45 (31.9)	96 (68.1)	141	**4.01 (2.41-6.66)**	8.56e-08	3.65 (2.48-5.38)
Gastric non-cardia [C16.1-C16.6]	55 (50.5)	54 (49.5)	109	**1.66 (0.95-2.91)**	0.074	1.69 (1.09-2.6)
Colorectum [C18-C20]	1713 (55.8)	1355 (44.2)	3068	**1.2 (1.07-1.33)**	0.001	1.22 (1.12-1.33)
Colon [C18]	1242 (59)	863 (41)	2105	**1.02 (0.9-1.16)**	0.757	1.04 (0.94-1.15)
Rectum [C19/C20]	482 (49.4)	494 (50.6)	976	**1.65 (1.37-2)**	2.09e-07	1.7 (1.47-1.96)
Anus [C21]	100 (79.4)	26 (20.6)	126	**0.29 (0.16-0.52)**	3.96e-05	0.41 (0.26-0.64)
Liver [C22]	146 (49.5)	149 (50.5)	295	**1.82 (1.29-2.56)**	0.001	1.91 (1.48-2.47)
Gallbladder [C23]	58 (75.3)	19 (24.7)	77	[insufficient data]	[insufficient data]	0.55 (0.31-0.97)
Pancreas [C25]	384 (58.3)	275 (41.7)	659	**0.98 (0.78-1.23)**	0.852	1.04 (0.87-1.25)
Lung [C34]	1190 (60.7)	769 (39.3)	1959	**0.95 (0.83-1.09)**	0.45	0.93 (0.84-1.03)
Lung adenocarcinoma [C34]	559 (65.9)	289 (34.1)	848	**0.72 (0.58-0.88)**	0.002	0.72 (0.62-0.84)
Lung squamous cell carcinoma [C34]	188 (49.7)	190 (50.3)	378	**1.45 (1.07-1.96)**	0.015	1.41 (1.14-1.75)
Lung small cell carcinoma [C34]	102 (58.3)	73 (41.7)	175	**1.24 (0.79-1.93)**	0.351	1.19 (0.86-1.64)
Melanoma [C43]	1041 (59.7)	703 (40.3)	1744	**0.97 (0.84-1.12)**	0.651	0.97 (0.86-1.09)
Breast [C50]	6603 (99.6)	28 (0.4)	6631	**0.006 (0.004-0.008)**	1.40e-159	0.005 (0.004-0.007)
Kidney [C64-65]	396 (45.3)	479 (54.7)	875	**1.67 (1.36-2.03)**	5.43e-07	1.77 (1.51-2.09)
Bladder [C67]	180 (30.6)	409 (69.4)	589	**3.61 (2.82-4.64)**	4.65e-24	3.47 (2.85-4.24)
Thyroid [C73]	212 (78.5)	58 (21.5)	270	**0.36 (0.24-0.53)**	3.65e-07	0.36 (0.26-0.49)
Lymphatic and haematopoietic [C81-C96]	1466 (54)	1251 (46)	2717	**1.2 (1.07-1.34)**	0.002	1.22 (1.11-1.33)

	Women (%)	Men (%)	Total count	**HR (95% CI)**	P value	Main analysis results*
Hodgkin lymphoma [C81]	47 (54.7)	39 (45.3)	86	[insufficient data]	[insufficient data]	1.29 (0.77-2.17)
Non-Hodgkin lymphoma [C82-C85]	724 (56.8)	550 (43.2)	1274	**1 (0.85-1.18)**	0.967	1.04 (0.91-1.19)
Multiple myeloma [C90 & C88]	294 (52.3)	268 (47.7)	562	**1.39 (1.09-1.79)**	0.008	1.35 (1.10-1.65)
Leukaemia [C91-C95]	395 (50.4)	389 (49.6)	784	**1.4 (1.14-1.73)**	0.002	1.43 (1.21-1.69)

Note: This sensitivity analysis was performed on a multivariable-adjusted model that included cancer-specific, and baseline set of covariates. Covariate selection has been described in text and supplementary tables. Cancer endpoints were excluded from the analysis if total counts were less 100. **301,509** participants self-reported as light drinkers in the cohort, meaning they consumed <1g/d to <20g/d of alcohol (grams/day). Of these 20,286 participants had malignant cancers, with 14,037 cases (69.2%) in women.

*The main analyses results for all participants (HRs and 95% CIs from multivariable-adjusted models with cancer specific covariates) are also shown for comparison.

Supplementary Table 5: Cancer specific multivariable-adjusted Cox proportional hazards models comparing risk of cancer incidence in men versus women in the highest qualification attainment stratum (Prof Q/NVQ/HND/HNC/Degree or other professional qualifications) in the UK Biobank.

	Women (%)	Men (%)	Total count	**HR (95% CI)**	P value	Main analysis results*
Oral cavity [C00-C14]	178 (34.5)	338 (65.5)	516	**1.93 (1.48-2.52)**	1.28e-06	2.06 (1.69-2.51)
Oesophagus [C15]	126 (24.5)	388 (75.5)	514	**2.92 (2.21-3.87)**	7.17e-14	2.89 (2.36-3.55)
Oesophageal adenocarcinoma [C15]	53 (14.6)	310 (85.4)	363	**6.27 (4.32-9.09)**	3.94e-22	5.45 (4.18-7.12)
Oesophageal squamous cell carcinoma [C15]	65 (52)	60 (48)	125	**0.6 (0.35-1.02)**	0.061	0.81 (0.55-1.2)
Stomach [C16]	89 (30.6)	202 (69.4)	291	**1.94 (1.35-2.79)**	0.000328	2.34 (1.83-2.97)
Gastric cardia [C16.0]	26 (18.6)	114 (81.4)	140	**4.04 (2.28-7.16)**	1.63e-06	3.65 (2.48-5.38)
Gastric non-cardia [C16.1-C16.6]	35 (39.8)	53 (60.2)	88	[insufficient data]	[insufficient data]	1.69 (1.09-2.6)
Colorectum [C18-C20]	1217 (40.9)	1761 (59.1)	2978	**1.14 (1.02-1.28)**	0.02	1.22 (1.12-1.33)
Colon [C18]	853 (43.7)	1099 (56.3)	1952	**1 (0.87-1.14)**	0.973	1.04 (0.94-1.15)
Rectum [C19-C20]	371 (35.7)	669 (64.3)	1040	**1.5 (1.24-1.81)**	3.51e-05	1.7 (1.47-1.96)
Anus [C21]	66 (61.1)	42 (38.9)	108	**0.44 (0.24-0.79)**	0.006	0.41 (0.26-0.64)
Liver [C22]	88 (30.7)	199 (69.3)	287	**1.83 (1.26-2.64)**	0.001	1.91 (1.48-2.47)
Gallbladder [C23]	31 (60.8)	20 (39.2)	51	[insufficient data]	[insufficient data]	0.55 (0.31-0.97)
Pancreas [C25]	284 (44.3)	357 (55.7)	641	**0.95 (0.75-1.2)**	0.656	1.04 (0.87-1.25)
Lung [C34]	709 (47)	800 (53)	1509	**0.92 (0.79-1.07)**	0.284	0.93 (0.84-1.03)
Lung adenocarcinoma [C34]	355 (52.5)	321 (47.5)	676	**0.69 (0.55-0.87)**	0.002	0.72 (0.62-0.84)
Lung squamous cell carcinoma [C34]	96 (33.6)	190 (66.4)	286	**1.57 (1.1-2.26)**	0.014	1.41 (1.14-1.75)
Lung small cell carcinoma [C34]	58 (43.9)	74 (56.1)	132	**1.21 (0.71-2.04)**	0.485	1.19 (0.86-1.64)
Melanoma [C43]	763 (47.5)	844 (52.5)	1607	**0.87 (0.75-1.01)**	0.076	0.97 (0.86-1.09)
Breast [C50]	4892 (99.3)	36 (0.7)	4928	**0.006 (0.004-0.008)**	6.7e-203	0.005 (0.004-0.007)
Kidney [C64-65]	238 (32.9)	485 (67.1)	723	**1.82 (1.45-2.29)**	2.11e-07	1.77 (1.51-2.09)

	Women (%)	Men (%)	Total count	**HR (95% CI)**	P value	Main analysis results*
Bladder [C67]	97 (17.5)	458 (82.5)	555	**3.93 (2.95-5.25)**	1.47e-20	3.47 (2.85-4.24)
Thyroid [C73]	156 (70.3)	66 (29.7)	222	**0.36 (0.24-0.55)**	1.92e-06	0.36 (0.26-0.49)
Lymphatic and haematopoietic [C81-C96]	1019 (41.4)	1443 (58.6)	2462	**1.18 (1.04-1.33)**	0.008	1.22 (1.11-1.33)
Hodgkin lymphoma [C81]	31 (45.6)	37 (54.4)	68	[insufficient data]	[insufficient data]	1.29 (0.77-2.17)
Non-Hodgkin lymphoma [C82-85]	504 (43.3)	660 (56.7)	1164	**1.07 (0.9-1.27)**	0.467	1.04 (0.91-1.19)
Multiple myeloma [C90 & C88]	226 (42.3)	308 (57.7)	534	**1.23 (0.95-1.6)**	0.113	1.35 (1.10-1.65)
Leukaemia [C91-C95]	251 (37)	427 (63)	678	**1.33 (1.06-1.68)**	0.015	1.43 (1.21-1.69)

Note: This sensitivity analysis was performed on a multivariable-adjusted model that included cancer-specific, and baseline set of covariates. Covariate selection has been described in text and supplementary tables. Cancer endpoints were excluded from the analysis if total counts were less than 100. **279,479** participants (51.3% women) were categorized in the highest qualification stratum based on their self-reports and it included individuals with 'College or university degree', 'NVQ or HND or HNC or equivalent', 'Other professional qualifications e.g.: nursing, teaching'. Within this strata, 17,392 participants had malignant cancers, with 9,953 cases (57.2%) in women.

* The main analyses results for all participants (HRs and 95% CIs from multivariable-adjusted models with cancer specific covariates) are also shown for comparison.

Supplementary Table 6: Cox proportional hazards models comparing risk of cancer incidence in men versus women additional adjustment for non-linear height (height^2) added to the model.

	HR (95% CI)	P value	Main analysis results*
Oral cavity [C00-C14]	**2.02 (1.66-2.47)**	4.07e-12	2.06 (1.69-2.51)
Oesophagus [C15]	**2.74 (2.22-3.38)**	1.03e-20	2.89 (2.36-3.55)
Oesophageal adenocarcinoma [C15]	**5.02 (3.78-6.67)**	8.98e-29	5.45 (4.18-7.12)
Oesophageal squamous cell carcinoma [C15]	**0.81 (0.55-1.19)**	0.291	0.81 (0.55-1.2)
Stomach [C16]	**2.22 (1.74-2.84)**	1.82e-10	2.34 (1.83-2.97)
Gastric cardia [C16.0]	**3.73 (2.48-5.62)**	2.87e-10	3.65 (2.48-5.38)
Gastric non-cardia [C16.1-C16.6]	**1.58 (1.03-2.42)**	0.036	1.69 (1.09-2.6)
Colorectum [C18-C20]	**1.22 (1.12-1.32)**	4.17e-06	1.22 (1.12-1.33)
Colon [C18]	**1.04 (0.94-1.15)**	0.484	1.04 (0.94-1.15)
Rectum [C19-C20]	**1.69 (1.46-1.96)**	1.92e-12	1.7 (1.47-1.96)
Anus [C21]	**0.42 (0.27-0.66)**	1.44e-04	0.41 (0.26-0.64)
Liver [C22]	**1.89 (1.46-2.46)**	1.78e-06	1.91 (1.48-2.47)
Gallbladder [C23]	**0.56 (0.32-1.00)**	0.049	0.55 (0.31-0.97)
Pancreas [C25]	**1.05 (0.88-1.25)**	0.606	1.04 (0.87-1.25)
Lung [C34]	**0.93 (0.84-1.02)**	0.134	0.93 (0.84-1.03)
Lung adenocarcinoma [C34]	**0.72 (0.62-0.84)**	2.49e-05	0.72 (0.62-0.84)
Lung squamous cell carcinoma [C34]	**1.41 (1.14-1.76)**	0.002	1.41 (1.14-1.75)
Lung small cell carcinoma [C34]	**1.18 (0.85-1.62)**	0.316	1.19 (0.86-1.64)
Melanoma [C43]	**0.97 (0.86-1.09)**	0.567	0.97 (0.86-1.09)
Breast [C50]	**0.005 (0.004-0.007)**	6.30e-302	0.005 (0.004-0.007)
Kidney [C64-65]	**1.8 (1.52-2.13)**	8.23e-12	1.77 (1.51-2.09)
Bladder [C67]	**3.78 (3.05-4.67)**	1.33e-34	3.47 (2.85-4.24)
Thyroid [C73]	**0.36 (0.26-0.5)**	1.28e-09	0.36 (0.26-0.49)
Lymphatic and haematopoietic [C81-C96]	**1.21 (1.11-1.33)**	3.60e-05	1.22 (1.11-1.33)
Hodgkin lymphoma [C81]	**1.24 (0.75-2.03)**	0.397	1.29 (0.77-2.17)
Non-Hodgkin lymphoma [C82-85]	**1.03 (0.9-1.18)**	0.635	1.04 (0.91-1.19)
Multiple myeloma [C90 & C88]	**1.37 (1.12-1.68)**	0.003	1.35 (1.10-1.65)
Leukaemia [C91-C95]	**1.43 (1.2-1.69)**	5.02e-05	1.43 (1.21-1.69)

Note: Analyses are adjusted for ethnicity, qualifications, height, height^2, BMI, smoking (status+intensity), alcohol consumption, and cancer specific risk factors, and stratified for age group, Townsend deprivation index, and region.

*The main analyses results for all participants (HRs and 95% CIs from multivariable-adjusted models with cancer specific covariates) are also shown for comparison.

Supplementary Table 7: Cox proportional hazards model comparing risk of cancer incidence in men versus women with additional adjustment for physical activity (METS/week)*.

	**HR (95% CI)**	P value	Main analysis results*
Oral cavity [C00-C14]	**2.12 (1.74-2.57)**	5.23e-14	2.06 (1.69-2.51)
Oesophagus [C15]	**2.91 (2.37-3.57)**	1.34e-24	2.89 (2.36-3.55)
Oesophageal adenocarcinoma [C15]	**5.47 (4.19-7.14)**	8.16e-36	5.45 (4.18-7.12)
Oesophageal squamous cell carcinoma [C15]	**0.86 (0.59-1.27)**	0.458	0.81 (0.55-1.2)
Stomach [C16]	**2.3 (1.81-2.93)**	1.39e-11	2.34 (1.83-2.97)
Gastric cardia [C16.0]	**3.62 (2.46-5.33)**	6.52e-11	3.65 (2.48-5.38)
Gastric non-cardia [C16.1-C16.6]	**1.69 (1.09-2.6)**	0.018	1.69 (1.09-2.6)
Colorectum [C18-C20]	**1.22 (1.13-1.33)**	1.91e-06	1.22 (1.12-1.33)
Colon [C18]	**1.04 (0.94-1.15)**	0.43	1.04 (0.94-1.15)
Rectum [C19/C20]	**1.7 (1.47-1.97)**	4.40e-13	1.7 (1.47-1.96)
Anus [C21]	**0.41 (0.26-0.64)**	8.55e-05	0.41 (0.26-0.64)
Liver [C22]	**1.94 (1.5-2.5)**	4.50e-07	1.91 (1.48-2.47)
Gallbladder [C23]	**0.57 (0.32-1.02)**	0.058	0.55 (0.31-0.97)
Pancreas [C25]	**1.03 (0.87-1.24)**	0.707	1.04 (0.87-1.25)
Lung [C34]	**0.94 (0.85-1.04)**	0.243	0.93 (0.84-1.03)
Lung adenocarcinoma [C34]	**0.72 (0.62-0.84)**	2.94e-05	0.72 (0.62-0.84)
Lung squamous cell carcinoma [C34]	**1.45 (1.17-1.8)**	6.50e-04	1.41 (1.14-1.75)
Lung small cell carcinoma [C34]	**1.21 (0.88-1.67)**	0.244	1.19 (0.86-1.64)
Melanoma [C43]	**0.96 (0.86-1.09)**	0.541	0.97 (0.86-1.09)
Breast [C50]	**0.005 (0.004-0.007)**	0.00e+00	0.005 (0.004-0.007)
Kidney [C64-65]	**1.78 (1.51-2.1)**	7.33e-12	1.77 (1.51-2.09)
Bladder [C67]	**3.46 (2.84-4.22)**	1.67e-34	3.47 (2.85-4.24)
Thyroid [C73]	**0.36 (0.26-0.49)**	4.76e-10	0.36 (0.26-0.49)
Lymphatic and haematopoietic [C81-C96]	**1.22 (1.11-1.34)**	2.16e-05	1.22 (1.11-1.33)
Hodgkin lymphoma [C81]	**1.29 (0.77-2.17)**	0.328	1.29 (0.77-2.17)
Non-Hodgkin lymphoma [C82-85]	**1.04 (0.91-1.19)**	0.569	1.04 (0.91-1.19)
Multiple myeloma [C90 & C88]	**1.36 (1.11-1.66)**	0.003	1.35 (1.10-1.65)
Leukaemia [C91-C95]	**1.43 (1.21-1.69)**	3.85e-05	1.43 (1.21-1.69)

Note: Physical activity was self-reported in the UK Biobank; participants answered questions about their physical activity levels at recruitment. Based on IPAQ guidelines, we categorized participants' physical activity levels (based on their metabolic equivalent (METS) per week) as low, moderate, high, and missing/unknown. The models adjusted for ethnicity, qualifications, height, BMI, smoking (status+intensity), alcohol consumption, physical activity levels, and cancer specific risk factors, and stratified by age group, Townsend deprivation index, and region.

*The main analysis results (HRs and 95% CIs from multivariable-adjusted models with cancer specific covariates) are shown for comparison.

Additional references

1. Townsend, P., Phillimore, P. & Beattie, A. Health and Deprivation. (Routledge, London, 2023). doi:10.4324/9781003368885.
2. Craig, C. L. *et al.* International physical activity questionnaire: 12-country reliability and validity. *Med Sci Sports Exerc* **35**, 1381–95 (2003).
3. Papier, K. *et al.* Meat consumption and risk of 25 common conditions: outcome-wide analyses in 475,000 men and women in the UK Biobank study. *BMC Med* **19**, 53 (2021).
4. Perez-Cornago, A. *et al.* Prospective investigation of risk factors for prostate cancer in the UK Biobank cohort study. *Br J Cancer* **117**, 1562–1571 (2017).
